# XXVII Reunión Anual de la Sociedad Canaria de Neurología (SOCANE). Libro de Comunicaciones

**DOI:** 10.31083/RN53020

**Published:** 2026-08-25

**Caso de Leucoencefalopatía Posterior Reversible**

Leonor Pinta Condor^1^, Noemi Bravo Alvarado^1^, Daniel Escáneo Otero^1^, Rayco Jiménez Bolaños^1^, Arminda Ruano Hernández^1^, Idaira Martín Santana^1^, Raúl Amela Peris^1^

^1^Servicio de Neurología, Complejo Hospitalario Universitario Insular Materno-Infantil (CHUIMI) de Gran Canaria, 35016 Las Palmas de Gran Canaria, España

**Introducción**: La leucoencefalopatía posterior reversible es un síndrome clínico-radiológico, causado por daño endotelial secundario a hipertensión. Se manifiesta con cefalea, confusión, y convulsiones; y tiene hallazgos característicos en neuroimagen. **Material y Método**: Caso clínico. **Resultados**: Varón de 53 años, hipertenso sin tratamiento. Acude al servicio de urgencias por convulsiones, confusión, cefalea, afasia e hipertensión, precedido de febrícula. En los exámenes complementarios se encontró: leucocitosis con neutrofilia, líquido cefalorraquídeo (LCR) con hiperproteinorraquia, sin células, y lactato elevado; en la Tomografía Computarizada (TC) aparece hemorragia subaracnoidea (HSA) aguda en surco frontal cortical izquierdo. Inicialmente se trata como una encefalitis herpética y se inicia aciclovir, levetiracetam y clonazepam. En el electroencefalograma (EEG) se objetiva encefalopatía moderada sin actividad epiléptica. En reacción en cadena de la polimerasa (PCR) de LCR se descarta etiología vírica; y tras el ajuste del tratamiento antihipertensivo se evidencia mejoría progresiva del lenguaje, no recurren las crisis, y en el EEG de control desaparece la encefalopatía. Se completó estudio con resonancia magnética (RM) Cerebral que muestra lesiones occipitales bilaterales, con afectación de la sustancia blanca periventricular y yuxtacortical, compatibles con encefalopatía posterior reversible. **Conclusiones**: Ante un paciente con el contexto clínico adecuado (en particular hipertensión, terapia inmunosupresora, enfermedad renal) con cefalea, confusión y convulsiones, se debe considerar el diagnóstico de leucoencefalopatía posterior reversible, que tiene una neuroimagen característica y una buena evolución clínica con el tratamiento antihipertensivo.

**De la Gonalgia al Singulto: A Propósito de un Caso**

Vanessa Pallarés Santos^1^, María José Hernández García^1^, Paloma Isabel Dupuy Oria^1^, Alba Elisa Bartolomé Yumar^1^, Ismael Naim Owrang Calvo^1^, Marcos Millet Oval^1^, Sofía del Águila Romero^1^, David Alejandro Padilla León^1^, Cristina Croissier Elías^1^, José Antonio Rojo Aladro^1^

^1^Servicio de Neurología, Hospital Universitario de Canarias, 38320 San Cristóbal de La Laguna, España

**Introducción**: El hipo se define como contracciones espasmódicas intermitentes del diafragma y los músculos intercostales que provocan el cierre brusco de la glotis, produciendo un sonido característico. Suele ser un fenómeno benigno y sin trascendencia patológica, aunque también puede ser consecuencia de enfermedades digestivas o intracraneales graves o al uso de fármacos. **Material y Método**: Caso clínico. **Resultados**: Presentamos un varón de 37 años, sin antecedentes de interés salvo latigazo cervical el mes previo, que acude por hipo de más de 24 horas de evolución, acompañado de episodio autolimitado de mareo y alteración sensitiva hemifacial izquierda. La exploración mostró disestesia parcheada en hemicara izquierda (territorio V1) y en cara interna de miembro inferior izquierdo, con hiperreflexia global. La neuroimagen (resonancia magnética [RM] cráneo/cervical y angioRM) y los estudios analíticos estuvieron dentro de la normalidad. El episodio de hipo cesó tras administración de una dosis única de metoclopramida. A la anamnesis dirigida, el paciente refirió administración intraarticular de dexametasona y anestésico local previo al inicio de la sintomatología. **Conclusiones**: Los corticosteroides son el grupo farmacológico que más se asocia al hipo persistente (>48 h). Su aparición tras infiltraciones de corticoesteroides tanto a nivel espinal como articular es una complicación muy poco frecuente, pero ya comunicada por otros autores. Aunque el mecanismo fisiopatológico es desconocido, es mucho más frecuente en varones y se han descrito recurrencias al repetir la infiltración, incluso años después.

**Desmontando Mitos Sobre la Esclerosis Múltiple: Impacto del Nivel de Información en la Autoestima de los Pacientes**

Jorge Cegarra Sánchez^1^, Alicia López Santana^1^, Juan Francisco García Granado^1^, Juan Rodríguez Santana^1^, Alejandro Relloso de la Fuente^1^, María del Carmen Pérez Vieitez^1^, Ayoze González Hernández^1^

^1^Servicio de Neurología, Hospital Universitario de Gran Canaria Doctor Negrín, 35010 Las Palmas de Gran Canaria, España

**Introducción**: La esclerosis múltiple (EM) es una enfermedad neurológica crónica cuya percepción y manejo se ven afectados por mitos persistentes. Este estudio analiza cómo la desinformación sobre aspectos clave (epidemiología, etiología, contagiosidad, carácter hereditario, sintomatología, papel de la actividad física, concepción y gravedad) influye en la autoestima percibida de los pacientes. **Material y Método**: Se realizó un estudio transversal con 75 pacientes diagnosticados de EM en Murcia y Gran Canaria. Los participantes completaron un cuestionario de 9 preguntas (escala 1–7) para evaluar su conocimiento sobre la enfermedad y la escala de autoestima de Rosenberg. Los datos se analizaron con el software estadístico SPSS de IBM, utilizando correlaciones para determinar significación estadística. **Resultados**: Aunque el nivel general de conocimiento fue adecuado, destacó el mito del «carácter hereditario» (creído por >50% de los pacientes), a pesar de la influencia genética no determinante en la EM. La consistencia interna de las creencias erróneas fue alta (α Cronbach = 0,801). Los pacientes con mayor información mostraron mejor autoestima percibida, con una correlación significativa (β = 0,319; *p* = 0,005). **Conclusiones**: Los mitos sobre la EM perpetúan desinformación que impacta negativamente en los pacientes y su entorno. Persisten errores, a destacar confundir predisposición genética con herencia, posiblemente por fallos en la comunicación médico-paciente. La educación basada en evidencia emerge como estrategia clave para empoderar al paciente y mejorar la autoestima, integrando estos aspectos en el manejo integral de la enfermedad.

**Epilepsia Mioclónica Progresiva por Mutación de Novo en Gen NUS1. A Propósito de un Caso**

Daniel Cardona Reyes^1^, Mercé Falip Centellas^1^, Jacint Salá-Padró^1^, Gema Hernández Pérez^1^, Arminda Ruano Hernánde^1^, Idaira Martín Santana^1^, Daniel Escáneo Otero^1^, Leonor Pinta Cóndor^1^, Noemi Bravo Alvarado^1^, Raúl Amela Perisúl^1^

^1^Servicio de Neurología, Complejo Hospitalario Universitario Insular Materno-Infantil (CHUIMI) de Gran Canaria, 35016 Las Palmas de Gran Canaria, España

**Introducción**: Las epilepsias mioclónicas progresivas (PME) son un grupo diverso de síndromes que se caracterizan por presentar una combinación de mioclonías, crisis epilépticas y deterioro neurológico progresivo. En la actualidad se conocen casi 50 alteraciones genéticas responsables, y se siguen descubriendo asociaciones con genes no clásicamente relacionados con PME. **Material y Métodos**: Presentamos el caso de una paciente con fenotipo sugestivo de PME con mutación en el gen *NUS1*. **Resultados**: Mujer de 43 años sin antecedentes relevantes con desarrollo psicomotor normal hasta los 8 años, cuando comienza con mioclonías de acción asociadas a dificultades para el aprendizaje. A partir de los 13 años comienza con temblor, dificultades para correr y crisis de ausencias, con empeoramiento psicomotor progresivo posterior. A los 20 años presenta mioclonías distales continuas en cuatro extremidades, crisis generalizadas y alteración psiquiátrica. Durante el estudio diagnóstico se realizaron varios electroencefalograma (EEG, con alteración del ritmo de base y elementos epileptiformes frecuentes), además de pruebas de neuroimagen y analíticas que no mostraron hallazgos concluyentes. Se realizó un panel genético de PME sin resultados, por lo que se decidió ampliar mediante HPOs (*Human phenotype ontology*), objetivándose mutación en el gen *NUS1*, descrito por primera vez en 2014, y que explica el espectro clínico de la paciente. **Conclusiones**: A pesar de que las PME son individualmente raras, en conjunto suponen un porcentaje importante del complejo mioclonías-epilepsia, y su diagnóstico de precisión nos permitirá desarrollar nuevas formas de tratamiento. Por tanto, es indispensable seguir ampliando el screening genético con nuevos genes como el *NUS1*.

**Estudio Exploratorio de Síndrome de Neurotoxicidad Asociada a Células Inmunoefectoras (ICANS) en Terapia CAR-T en Centro de Referencia de Canarias**

Alicia López Santana^1^, Leslie González Pinedo^2^, María del Mar Perera Álvarez^2^, Hugo Luzardo Henríquez^2^, Santiago Díaz Nicolás^1^, María del Carmen Pérez Vieitez^1^, Ayoze González Hernández^1^, Juan Francisco García Granado^1^, Jorge Cegarra Sánchez^1^, Juan Rodríguez Santana^1^, Alejandro Relloso de la Fuente^1^

^1^Servicio de Neurología, Hospital Universitario de Gran Canaria Doctor Negrín, 35010 Las Palmas de Gran Canaria, España

^2^Servicio de Hematología, Hospital Universitario de Gran Canaria Doctor Negrín, 35010 Las Palmas de Gran Canaria, España

**Introducción**: La terapia de linfocitos T con Receptor de Antígeno Quimérico (CAR-T) implica la modificación genética de linfocitos T para reconocer y destruir células tumorales. Sin embargo, puede generar toxicidades agudas, como el síndrome de liberación de citocinas (SLC) y/o Síndrome de Neurotoxicidad Asociada a Células Inmunoefectoras (ICANS). Este estudio explora la incidencia y factores asociados al desarrollo de ICANS en pacientes con neoplasias hematológicas tratados con terapia CAR-T. **Material y Métodos**: Estudio descriptivo de una muestra de 47 pacientes tratados con terapia CAR-T en centro de referencia de Canarias desde su implementación en el año 2020 hasta febrero/2025. Se recoge en una base de datos tipos y carga tumoral, características clínicas, toxicidades agudas y evolución, y se describe su relación. **Resultados**: El 49% desarrolló ICANS, distribuidos en 48% casos precoces y 52% tardíos; con una gravedad variable (43% grado 1, 13% grado 2, 26% grado 3, 17.4% grado 4). Hasta un 82% presentaba alta carga tumoral previa al tratamiento, y un 9% comorbilidades neurológicas. Todos los casos de ICANS presentaron SLC (56% grado 1, 44% grado 2), y el 87% recibió al menos una dosis de Tocilizumab. En términos de evolución, hubo un 9% de mortalidad, mientras que en un 91% la toxicidad resultó reversible. **Conclusiones**: El estudio muestra una alta incidencia de neurotoxicidad, especialmente en aquellos con alta carga tumoral. La mayoría de los casos de ICANS se asociaron a SLC, y los grados severos conllevaron complicaciones graves e incluso mortalidad. Estos hallazgos resaltan la importancia de una vigilancia neurológica y manejo precoz, especialmente en pacientes con factores de riesgo identificables.

**Evolución de Pacientes con Parkinson Tras la Implantación de Estimulación Cerebral Profunda (DBS) en un Hospital de Tercer Nivel**

Alejandro Relloso de la Fuente^1^, Jesús de la Nuez González^1^, Alicia López Santana^1^, Juan Francisco García Granado^1^, Jorge Cegarra Sánchez^1^, Juan Rodríguez Santana^1^, María Jesús Alemany Rodríguez^1^, María del Carmen Pérez Vieitez^1^, Ayoze González Hernández^1^, Marta Morera Molina^2^, José Andrés Suárez Muñoz^1^, Sara Bisshopp Alfonso^2^

^1^Servicio de Neurología del Hospital Universitario de Gran Canaria Doctor Negrín, 35010 Las Palmas de Gran Canaria, España

^2^Servicio de Neurocirugía del Hospital Universitario de Gran Canaria Doctor Negrín, 35010 Las Palmas de Gran Canaria, España

**Introducción**: La enfermedad de Parkinson es una patología neurodegenerativa con afectación motora y no motora, que repercute en la funcionalidad del paciente. En sus primeras etapas la sintomatología puede ser controlada farmacológicamente, pero con la progresión de la enfermedad son necesarias otras técnicas. Entre ellas, la estimulación cerebral profunda (DBS) consiste en la colocación intracraneal de electrodos en el núcleo subtalámico/globo pálido conectados a un neuroestimulador subcutáneo. En este trabajo analizamos la evolución de los pacientes intervenidos de DBS en nuestro centro. **Material y Métodos**: Se incluyeron 114 pacientes a los que se les implantó el DBS entre noviembre de 2001 y agosto de 2022. Se les aplicó la escala UPDRS III previo al tratamiento, a los 6 meses y a los 12 meses posteriores al mismo. Además, se registraron los efectos secundarios más comunes a consecuencia de la terapia. **Resultados**: Tras la implantación del DBS, se observó una disminución media de 3.28 puntos en la puntuación de la escala UPDRS III a los 6 meses (SD ± 6.83; *p* = 0.11) y de 2.39 puntos posteriormente (SD ± 6.08; *p* = 0.14). Se registraron efectos adversos en 20 de los pacientes a los 6 meses y en sólo 1 a los 12 meses, destacando mayoritariamente las discinesias y el empeoramiento en la marcha. **Conclusiones**: Los resultados evidencian que en nuestra población se ha producido una mejoría clínica relevante tras el tratamiento. Sin embargo, los resultados no han alcanzado la significación estadística, por lo que sería conveniente realizar más estudios ampliando el tamaño muestral para confirmar estos hallazgos.

**Ictus Isquémico Bihemisférico Secundario a Síndrome de Eagle**

Ismael Naim Owrang Calvo^1^, María José Hernández García^1^, David Alejandro Padilla León^1^, Alba Elisa Bartolomé Yumar^1^, Marcos Millet Oval^1^, Sofía del Águila Romero^1^, Vanessa Pallarés Santos^1^, Paloma Isabel Dupuy Oria^1^, José Antonio Rojo Aladro^1^

^1^Servicio de Neurología, Hospital Universitario de Canarias, 38320 San Cristóbal de La Laguna, España

**Introducción**: Presentar un caso de ictus isquémico de etiología inhabitual secundario a un síndrome de Eagle. **Material y Métodos**: Revisión de la historia clínica del paciente. **Resultados**: Mujer de 69 años de edad, dislipémica y diabética, que ingresó en la Unidad de Ictus por episodios de repetición de alteración del lenguaje, sin dolor cervical ni disfagia asociada, a lo largo de 24 h. En la exploración presentó una afasia mixta de predominio motor con disartria grave sin déficit motor asociado. En la analítica destacó un perfil metabólico subóptimo. La RM cerebral objetivó infartos agudos en el hemisferio izquierdo y territorio dependiente de la arteria cerebral anterior derecha. El angio-TC reveló un trombo intraluminal asociado a disección focal de la arteria carótida interna secundaria a una compresión por la apófisis estiloides izquierda por elongación de la misma. Asimismo, se evidenció un segmento A1 derecho hipoplásico condicionando el flujo de la arteria cerebral anterior derecha, dependiendo esta de la circulación izquierda. Se decidió anticoagulación con heparina de bajo peso molecular (HBPM) y tratamiento quirúrgico programado por parte del S. C. Maxilofacial. **Conclusiones**: Este caso ayuda a recordar el manejo de una de las etiologías inhabituales poco comunes del ictus isquémico, el Síndrome de Eagle estilocarotídeo. Además, como complicación, la paciente presentó una disección carotídea con trombo intraluminal asociado, resolviéndose este último tras inicio de tratamiento anticoagulante.

**Ictus Isquémico Tras la Embolización de Arterias Bronquiales**

Magnamara Oliva Martín^1^, Jeidy Marcela Henao Ramírez^1^, María del Rosario Ríos Cejas^1^, María Teresa Florido Capilla^1^, Sandra Rodríguez Martín^1^, Alba Jiménez Barreto^1^

^1^Servicio de Neurología del Hospital Universitario Nuestra Señora de Candelaria (HUNSC), 38010 Santa Cruz de Tenerife, España

**Introducción**: La embolización de la arteria bronquial (EAB) es un procedimiento indicado para el tratamiento sintomático de la hemoptisis masiva o recurrente. Puede asociarse con complicaciones neurológicas, como el ictus isquémico y la isquemia medular, con una incidencia estimada de <1% y 0,2%, respectivamente. Entre los mecanismos implicados se incluyen shunts entre arterias bronquiales y la circulación sistémica (subclavia, vertebral o intercostales), reflujo retrógrado del material embolizante y variantes anatómicas (como el origen ectópico de arterias bronquiales desde las vertebrales o espinales). **Material y Métodos**: Se presenta una revisión de dos casos clínicos recogidos en 2024, de pacientes con hemoptisis que se sometieron a EAB izquierdas. **Resultados**: Caso 1. Mujer de 52 años, fumadora. Veinticuatro horas después de la EAB, presentó alteración del lenguaje y dismetría en las extremidades derechas. La resonancia magnética (RM) cerebral mostró lesiones isquémicas múltiples en cerebelo, territorio de las arterias perforantes basilares derechas, arterias cerebrales posteriores y arterias lenticuloestriadas izquierdas. La revisión de la arteriografía evidenció un shunt entre la arteria bronquial izquierda y la subclavia izquierda. El resto de los estudios complementarios fueron normales. Caso 2. Mujer de 55 años, fumadora. Inmediatamente después de la EAB desarrolló una paraparesia e hipoestesia de predominio izquierdo. La RM de columna mostró una lesión isquémica medular aguda desde el nivel T2 hasta T4. **Conclusiones**: La EAB, aunque es un procedimiento eficaz y seguro, puede asociarse con complicaciones neurológicas graves. La presencia de shunts, el reflujo de material embolizante y las variantes anatómicas vasculares constituyen factores clave en su fisiopatología.

**Mielopatía Actínica: Desafío Diagnóstico Cuatro Décadas Después**

Alba Elisa Bartolomé Yumar^1^, David Alejandro Padilla León^1^, Fernando Otón Sánchez^1^, María José Hernández García^1^, Ismael Naim Owrang Calvo^1^, Marcos Millet Oval^1^, Sofía del Águila Romero^1^, Paloma Isabel Dupuy Oria^1^, Vanessa Pallarés Santos^1^, José Antonio Rojo Aladro^1^

^1^Servicio de Neurología, Servicio de Oncología Radioterápica, Hospital Universitario de Canarias, 38320 San Cristóbal de La Laguna, España

**Introducción**: La mielopatía actínica es una complicación rara pero grave de la radioterapia. Existen tres formas según su cronología: aguda, temprana tardía y tardía, siendo esta última infrecuente y de peor pronóstico. Factores de riesgo incluyen dosis total de radiación superior a 45–50 Gy, fraccionamiento inadecuado, volumen irradiado, edad avanzada, entre otros. **Material y Métodos**: Caso clínico de una mielitis actínica tardía. **Resultados**: Varón de 57 años con antecedente de meduloblastoma tratado con radioterapia a los 15 años, quien consulta por debilidad progresiva en miembros inferiores (MMII) de tres semanas de evolución. En la exploración neurológica destaca una paraparesia espástica de MMII, hipopalestesia moderada bilateral con gradiente distal, abolición de sensibilidad artrocinética en MMII e hiperreflexia generalizada. Las primeras dos resonancias magnéticas (RM) no mostraron alteraciones. Estudios neurofisiológicos revelaron afectación del tracto corticoespinal y aumento de las latencias en los potenciales evocados visuales (PEV) somatosensoriales. Se descartaron causas autoinmunes, infecciosas, metabólicas, vasculares y paraneoplásicas. La evolución clínica fue desfavorable, con progresión a paraplejia y afectación esfinteriana. Una tercera RM mostró lesiones medulares compatibles con daño por radiación. Se inició tratamiento con oxígeno hiperbárico (39 sesiones), logrando estabilización clínica. Posteriormente, recibió rehabilitación intensiva (UMI) con recuperación funcional parcial. Actualmente, mantiene seguimiento ambulatorio con mejoría. **Conclusiones**: Se destaca la importancia de considerar la mielopatía actínica tardía como diagnóstico en pacientes con antecedentes de radioterapia, incluso décadas después. Las técnicas modernas de radioterapia reducen el riesgo, pero la vigilancia a largo plazo sigue siendo esencial. El oxígeno hiperbárico puede ofrecer beneficios en casos seleccionados, especialmente cuando se inicia en fases tempranas del deterioro neurológico.

**Stroke-Mimic de Territorio V-B: Hay Que Mirar Más Allá de la Receta Electrónica**

Paloma Isabel Dupuy Oria^1^, María José Hernández García^1^, Lucas Darío Iacampo Leiva^1^, David Alejandro Padilla León^1^, Vanessa Pallarés Santos^1^, Alba Elisa Bartolomé Yumar^1^, Ismael Naim Owrang Calvo^1^, Marcos Millet Oval^1^, Sofía del Águila Romero^1^, José Antonio Rojo Aladro^1^

^1^Servicio de Neurología, Hospital Universitario de Canarias, 38320 San Cristóbal de La Laguna, España

**Introducción**: Los stroke mimics (SM) son condiciones no vasculares que provocan déficits neurológicos superponibles al de los eventos cerebrovasculares, que pueden imitar disfunciones de cualquier topografía cerebral. Es fundamental realizar un adecuado diagnóstico diferencial de estas patologías, dadas las diferentes implicaciones terapéuticas y pronósticas de cada una de ellas. **Material y Métodos**: Caso clínico. **Resultados**: Varón de 61 años, con antecedentes personales de epilepsia desde 2019 en tratamiento con Carbamazepina 200 mg en cena, que ingresa por cuadro vertiginoso acompañado de nistagmus vertical en la supraversión y horizonto-rotatorio en miradas laterales extremas, con sutil pronación sin claudicación de mano izquierda. El paciente ingresa en la Unidad de Ictus, con estudio etiológico donde destaca la ausencia de lesiones agudas vasculares en RM y un perfil lipídico subóptimo, con resto de estudio analítico y neurovascular sin alteraciones. El paciente negaba cambios en su medicación habitual, pero al insistir en la anamnesis, confesó toma de 3 comprimidos de Carbamazepina 200 mg por dificultad para conciliar el sueño la noche previa. Se solicitaron niveles del fármaco (19,3 µg/mL; rango tóxico >10/12 µg/mL), con mejoría clínica y analítica tras suspender la medicación, por lo que se interpretó el cuadro como secundario a toxicidad por dicho medicamento. **Conclusiones**: Los SM deben estar incluidos dentro del diagnóstico diferencial, incluso en casos con síndromes clínicos más típicos. La toxicidad por carbamazepina puede manifestarse con ataxia, diplopía y oftalmoparesia, por lo que debe plantearse en el diagnóstico diferencial ante sospecha de ictus en territorio vertebro-basilar.

**Glioblastoma Multiforme Como Causa Infrecuente de Síndrome Corticobasal: Presentación de un Caso**

Daniel Escáneo Otero^1^, Laura Fernández Pérez^1^, Daniel Cardona Reyes^1^, Leonor Pinta Cóndor^1^, Noemi Bravo Alvarado^1^, Idaira Martín Santana^1^, Arminda Ruano Hernández^1^, Raúl Amela Peris^1^

^1^Servicio de Neurología, Complejo Hospitalario Universitario Insular Materno-Infantil de Las Palmas de Gran Canaria, 35016 Las Palmas de Gran Canaria, España

**Introducción**: El glioblastoma multiforme es el tumor cerebral primario más frecuente y agresivo en adultos. Su presentación clínica puede ser muy variable y simular patologías neurodegenerativas, retrasando el diagnóstico. Presentamos el caso de un varón inicialmente valorado por deterioro cognitivo y parkinsonismo, en quien finalmente se identificó un glioblastoma. **Material y Métodos**: Varón de 69 años, sin antecedentes de interés, que acude a consulta de neurología por un cuadro de dos años de evolución de deterioro cognitivo progresivo, alteraciones conductuales y parkinsonismo asimétrico de predominio izquierdo. Se objetivan además disgrafia, apraxia ideomotora, alteración de la sensibilidad cortical y temblor polimioclónico. Se inicia tratamiento con Donepezilo y Trazodona sin mejoría clínica, y se solicitan pruebas para estudio ambulatorio. **Resultados**: Meses después, el paciente acude a urgencias por instauración brusca de hemiparesia izquierda. En la TC craneal urgente se observa lesión hipodensa en ínsula derecha, confirmada posteriormente por RM como masa expansiva infiltrante. Se realiza biopsia estereotáxica, cuyo análisis anatomopatológico revela glioblastoma multiforme. Se descarta patología vascular aguda y se inicia tratamiento oncológico multidisciplinar. **Conclusiones**: El glioblastoma puede manifestarse con síntomas subagudos o progresivos, simulando enfermedades neurodegenerativas y parkinsonismos atípicos. La aparición súbita de déficits neurológicos focales debe hacer reconsiderar el diagnóstico inicial. La neuroimagen juega un papel clave en la identificación precoz de lesiones estructurales en pacientes con clínica neurológica atípica o progresiva.

**Encefalopatía Epiléptica y del Desarrollo Asociada a Síndrome del Doble Córtex y Lisencefalia por Mutación Patogénica en Gen DCX**

Juan Francisco García Granado^1^, Jesús González de la Aleja^2^, Vega Stride González^2^, María del Carmen Pérez Vieitez^1^, María Jesús Alemany Rodríguez^1^, Alicia López Santana^1^, Ayoze González Hernández^1^, Jorge Cegarra Sánchez^1^, Alejandro Relloso de la Fuente^1^, Juan Rodríguez Santana^1^

^1^Servicio de Neurología, Hospital Universitario de Gran Canaria Doctor Negrín, 35010 Las Palmas de Gran Canaria, España

^2^Servicio de Neurología, Hospital Universitario 12 de Octubre, 28041 Madrid, España

**Introducción**: Las variantes patogénicas en el gen de la doblecortina (DCX) causan lisencefalia y heterotopia subcortical laminar (HSL) con herencia ligada al X. La lisencefalia implica un fallo en la migración neuronal, asociado a discapacidad intelectual severa y epilepsia refractaria. En mujeres heterocigotas, el fenotipo más leve se conoce como síndrome del doble córtex (SDC). **Material y Métodos**: Presentamos el caso de una paciente con discapacidad intelectual moderada y epilepsia refractaria en contexto de encefalopatía epiléptica y del desarrollo asociado a SDC y lisencefalia de etiología genética por variante patogénica en el gen *DCX*. **Resultados**: Mujer de 25 años, con retraso del neurodesarrollo y episodios de desconexión del medio, desviación oculo-cefálica alternante y clúster de crisis atónicas tronco-cefálicas desde los 13 años de edad. Es diagnosticada de síndrome de Lennox Gastaut con evolución clínica desfavorable a pesar de politerapia anti-crisis. En electroencefalograma (EEG) se objetivó actividad epileptiforme intercrítica generalizada en forma de punta onda aguda asimétrica de predominio derecho, acentuada con la hiperventilación y suprimida parcialmente tras el cierre ocular (fixation-OFF). En RM craneal se constató heterotopia neuronal laminar en banda bihemisférica que ocupa la sustancia blanca con paquigiria, microcefalia y lisencefalia, así como focos heterotópicos periventriculares. Se realizó estudio genético, detectándose variante patogénica de novo *c.1009A>T*, p.(Lys337Ter), en el gen *DCX* con un patrón de herencia ligado al X. Se implantó terapia con estimulador del nervio vago con estabilidad clínica actual. **Conclusiones**: Este caso presenta una encefalopatía epiléptica genética con SDC refractaria por mutación en *DCX* y con mejoría clínica tras estimulación del nervio vago.

**Análisis de Características Clínicas y Etiologías del Ictus Isquémico en Paciente Joven en el Hospital Doctor Negrín en los Últimos 25 Años**

Juan Francisco García Granado^1^, María del Carmen Pérez Vieitez^1^, María Jesús Alemany Rodríguez^1^, Ayoze González Hernández^1^, Alicia López Santana^1^, Jorge Cegarra Sánchez^1^, Alejandro Relloso de la Fuente^1^, Juan Rodríguez Santana^1^

^1^Servicio de Neurología, Hospital Universitario de Gran Canaria Doctor Negrín (HUGCDN), 35010 Las Palmas de Gran Canaria, España

**Introducción**: El 10–15% de los ictus isquémicos ocurre en menores de 50 años, con una edad promedio en descenso en los últimos años. **Material y Métodos**: Estudio analítico observacional retrospectivo de la frecuencia de factores de riesgo vascular (FRV) y etiologías del ictus isquémico en jóvenes (18–50 años) atendidos en el HUGCDN en los últimos 25 años. Recogida de datos mediante FileMakerPro Neurología. Variables recogidas: sexo, edad, FRV clásicos, ictus y enfermedad coronaria previas, tipo de ictus isquémico (OCSP/TOAST) y NIHSS y mRS al ingreso y al alta. Se dividieron las etiologías inhabituales en 5 grupos: disección, enfermedades autoinmunes, enfermedades hematológicas, arteriopatías y embolias paradójicas por shunt derecha-izquierda. **Resultados**: n = 414 pacientes (edad media 43 años, 58,45% varones). La incidencia fue fluctuante (pico 2006–2012). El FRV más prevalente fue el tabaquismo (n = 236, 57%). El 61,1% de los pacientes de 41–50 años tenían 2 o más FRV (*p* < 0.001). La etiología predominante fue la inhabitual (35,02%), específicamente la disección (35,17%) y la embolia paradójica (33,1%), siendo estos más frecuentes en menores de 40 años, mientras que las etiologías aterotrombótica y lacunar predominaron en mayores de 40 (*p* = 0.004). El 87,7% de la muestra presentaron ictus leves al ingreso (NIHSS <12; mediana 3; rango intercuartílico 2.0–7.0), sobre todo los varones (*p* = 0.005), quienes presentaron un mejor pronóstico funcional al alta (*p* = 0.019). **Conclusiones**: La mayoría de los pacientes eran fumadores y presentaron ictus leves al ingreso, con predominio de etiologías inhabituales en menores de 40 años y mejor resultado funcional en varones.

**Pérdida de Visión Unilateral Transitoria Aislada Como Début de una Vasculitis: A Propósito de un Caso**

Marcos Millet Oval^1^, María José Hernández García^1^, David Alejandro Padilla León^1^, Sofía del Águila Romero^1^, Alba Elisa Bartolomé Yumar^1^, Ismael Naim Owrang Calvo^1^, Vanessa Pallarés Santos^1^, Paloma Isabel Dupuy Oria^1^, José Antonio Rojo Aladro^1^

^1^Servicio de Neurología, Complejo Hospitalario Universitario de Canarias, 38320 San Cristóbal de La Laguna, España

**Introducción**: La arteritis de células gigantes (ACG) o de la temporal es una vasculitis granulomatosa de arterias de mediano y gran calibre. Típicamente afecta a mayores de 50 años, y suele cursar con cefalea, claudicación mandibular, astenia y/o síntomas visuales tanto transitorios como permanentes. **Material y Métodos**: Presentamos un caso clínico. **Resultados**: Varón de 61 años, con antecedentes de exfumador, hipertensión arterial, nefrolitiasis y glaucoma. Acude por episodio de 20 minutos de pérdida de visión completa indolora por el ojo derecho tras esfuerzo físico. A la exploración, el paciente está asintomático (NIHSS: 0) y niega presentar cefalea, dolor ocular ni cervical. La RM no muestra alteraciones estructurales ni isquemia aguda. Los estudios neurovasculares descartan estenosis carotídea, destacando únicamente aumento de calibre de arterias temporales en secuencia fluid-attenuated inversion recovery (FLAIR). Los análisis muestran parámetros inflamatorios elevados (Velocidad de Sedimentación Globular 90 mm/h, Proteína C Reactiva 61 mg/dL) y anemia (Hb 10.9 g/dL) no conocidos, hallazgos sugestivos de ACG. La ecografía de arterias temporales mostró signo del halo y se programó biopsia, iniciándose corticoterapia. La ACG se confirmó histopatológicamente y se continuó tratamiento inmunosupresor (prednisona vo, posteriormente asociando adalimumab). Los análisis y la ecografía se habían normalizado a los 6 meses, sin nuevos síntomas deficitarios. **Conclusiones**: La afectación ocular ocurre en el 50% de los pacientes con ACG, siendo la pérdida visual unilateral irreversible la más común. De estos, 30% presentan previamente episodios de amaurosis fugax, por lo que incluso en ausencia de otros síntomas debemos incluir la ACG en el diagnóstico diferencial. El inicio de corticoterapia de forma temprana es fundamental para evitar secuelas visuales.

**Síndrome de Alarma Capsular No Capsular**

Marcos Millet Oval^1^, María José Hernández García^1^, Dionisio Miguel García Álvarez^1^, Sofía del Águila Romero^1^, Alba Elisa Bartolomé Yumar^1^, Ismael Naim Owrang Calvo^1^, Paloma Isabel Dupuy Oria^1^, Vanessa Pallarés Santos^1^, José Antonio Rojo Aladro^1^

^1^Servicio de Neurología, Complejo Hospitalario Universitario de Canarias, 38320 San Cristóbal de La Laguna, España

**Introducción**: El síndrome de alarma capsular (SAC) se define como tres o más episodios recurrentes, estereotipados y autolimitados de déficit motor y/o sensitivo afectando a cara, brazo o pierna, sin signos corticales, en un período de menos de 72 horas. Habitualmente se asocia a infartos capsulares, aunque puede localizarse en cualquier punto del tracto corticoespinal. **Material y Métodos**: Presentamos un caso clínico. **Resultados**: Varón de 46 años, con antecedentes de hipertensión arterial y obesidad. Acude por síndrome sensitivo-motor facio-braquio-crural derecho con disartria moderada de 1 hora de evolución (NIHSS 12). TC cráneo normal, con angioTC con dudoso defecto de repleción en origen de rama capsular de arteria cerebral media izquierda. Se indica fibrinolisis endovenosa, con mejoría progresiva hasta recuperación completa (NIHSS: 0). 2 horas después presenta empeoramiento neurológico (NIHSS 12, superponible) y se indica trombectomía mecánica de rescate, sin detectar oclusión en la arteriografía. Al finalizar el procedimiento el paciente está asintomático, pero 48 horas después presenta nuevo empeoramiento, con recuperación parcial espontánea. La RM muestra ictus agudo hemiprotuberancial izquierdo, sin oclusión ni estenosis en angioRM. Se inicia doble antiagregación, antihipertensivos e hipolipemiantes; presentando al alta disartria leve y sutil debilidad distal mano derecha (NIHSS:1). **Conclusiones**: Sólo el 50% de los SAC presentan lesión en cápsula, existiendo localizaciones atípicas como nuestro caso clínico. Esta entidad condiciona un alto riesgo de desarrollar un ictus (40–70%) a los 10 días; mayor que otra isquemia cerebral transitoria. La efectividad de la fibrinolisis es controvertida, aunque parece ser segura. La doble antiagregación podría reducir las fluctuaciones clínicas y mejorar el pronóstico funcional.

**Estatus Suprarrefractario Asociado a Anticuerpos Anti-SSA**

Noemi Bravo Alvarado^1^, Leonor Pinta Cóndor^1^, Daniel Escáneo Otero^1^, Arminda Ruano Hernández^1^, Idaira Martín Santana^1^, Raúl Amela Peris^1^

^1^Servicio de Neurología, Centro Hospitalario Universitario Insular Materno Infantil (CHUIMI), 35016 Las Palmas de Gran Canaria, España

**Introducción**: El estado epiléptico suprarrefractario (ESR) se define como la persistencia de actividad epiléptica de más de 24 horas a pesar del empleo de tratamiento de tercera línea, asociando una elevada morbimortalidad. Entre sus causas más conocidas se incluyen las encefalitis infecciosas, las alteraciones estructurales y los accidentes cerebrovasculares. No obstante, cada vez presenta mayor relevancia la etiología autoinmune, especialmente en pacientes jóvenes sin antecedentes médicos. **Material y Método**: Mujer de 34 años sin antecedentes personales de interés que acudió a urgencias por una crisis epiléptica de inicio focal motora con alteración progresiva del nivel de consciencia en contexto de un cuadro pseudogripal de 4 días. **Resultados**: Inicialmente se detectó un líquido cefalorraquídeo (LCR) con pleocitosis mononuclear e hiperproteinorraquia, orientando el diagnóstico como una encefalitis infecciosa, por lo que fue tratada con antibioterapia de amplio espectro asociando dexametasona. A pesar de lo cual se trasladó a la Unidad de Medicina Intensiva por empeoramiento del nivel de consciencia. Se amplió estudio con RM que mostró afectación simétrica en ganglios basales y tálamo y EEG con patrón delta Brush generalizada así como actividad epileptiforme. En autoinmunidad se detectaron niveles altos de anticuerpos anti‑antígeno soluble A (anti-SSA) iniciándose tratamiento con inmunoterapia. **Conclusiones**: Ante un caso clínico de ESR se debe plantear la posibilidad etiológica autoinmune, especialmente en pacientes jóvenes y sin patologías previas. La detección de anticuerpos como anti-SSA, anti‑receptor N‑metil‑D‑aspartato (anti-NMDAR), anti‑descarboxilasa del ácido glutámico 65 (anti-GAD65), anti‑leucina‑rico glioma inactivado 1 (anti-LGI1) o anti‑proteína asociada a contacto 2 (anti-CASPR2) es clave para poder instaurar de forma precoz un tratamiento inmunomodulador.

**Neuromielitis Óptica Secundaria a Tratamiento Inmunomodulador**

Noemi Bravo Alvarado^1^, Leonor Pinta Cóndor^1^, Daniel Escáneo Otero^1^, Pino López Méndez^1^, Arminda Ruano Hernández^1^, Idaira Martín Santana^1^, Raúl Amela Peris^1^

^1^Servicio de Neurología, Centro Hospitalario Universitario Insular Materno Infantil (CHUIMI), 35016 Las Palmas de Gran Canaria, España

**Introducción**: La neuromielitis óptica (NMO) es una enfermedad inflamatoria clásicamente inducida por mecanismos autoinmunes primarios. No obstante, existen formas secundarias provocadas de forma iatrogénica por fármacos inmunomoduladores como los inhibidores del factor de necrosis tumoral (anti-TNFα) o los inhibidores de check point (ICP). **Material y Método**: Mujer de 65 años con riesgo vascular y adenocarcinoma endometrioide grado 1 tratado con ICP. En este contexto presenta neumonitis inmunomediada grado 3 que se trata con anti-TNFα. Posteriormente ingresa en Oncología por dificultad respiratoria y hemiparesia derecha progresiva que evoluciona a tetraparesia. Se pautan corticoides e ingresa en medicina intensiva (UMI). **Resultados**: Estando en UMI se objetiva mielitis longitudinalmente extensa de C1-C3 y C6-D2. Se decide ventana de sedación. Al despertar la paciente no percibe luz y presenta tetrapléjica. Se realiza RM cerebral en ese momento donde destaca la afectación extensa de ambos nervios ópticos. En la punción lumbar: 7 células, proteínas de 49.3 mg/dL, interleucina-6 (IL-6) de 11.3 pg/mL, Bandas Oligoclonales negativas. Además, presenta linfopenia de 400 linfocitos/µL e hipogammaglobulinemia. Se pauta tratamiento con anti-IL6 y plasmaféresis. Ante falta de mejoría y la existencia de manifestación de voluntades anticipadas, se retiran medidas invasivas de soporte y la paciente fallece. **Conclusiones**: Este caso de NMO iatrogénica resalta la necesidad de una vigilancia estrecha ante síntomas neurológicos en pacientes que reciben terapias con fármacos inmunomoduladores.

**Encefalitis de Rasmussen: A Propósito de un Caso**

Saskia Vigni^1^, Alejandro Rodríguez Vallejo^1^, María Teresa Florido Capilla^1^, Sandra Rodríguez Martín^1^, Alba Jiménez Barreto^1^, María del Rosario Ríos Cejas^1^, Javier Crisóstomo Pardillo^1^, Desiré González Barrios^2^, Lucía Martin Viota^2^

^1^Servicio de Neurología, Hospital Universitario Nuestra Señora de Candelaria, 38010 Santa Cruz de Tenerife, España

^2^Servicio de Pediatría, Hospital Universitario Nuestra Señora de Candelaria, 38010 Santa Cruz de Tenerife, España

**Introducción**: La encefalitis de Rasmussen es una enfermedad neurológica poco frecuente que cursa con una inflamación crónica a nivel cerebral y asocia epilepsia focal farmacorresistente y déficits neurológicos. **Material y Método**: Mujer de 19 años, zurda, que debutó a los 4 años con crisis epilépticas de inicio focal. Tuvo un desarrollo psicomotor normal hasta el debut de epilepsia. Posteriormente presentó crisis focales recurrentes con evolución temprana a estatus epiléptico y asociación de hemiparesia. Se realizó neuroimagen que evidenció alteración de señal periventricular izquierdo y se inició tratamiento anticrisis. Reingresó meses más tarde por nuevo estatus, realizándose una tomografía por emisión de positrones (PET) que mostró hipometabolismo hemisférico izquierdo. Recibió inmunoterapia en asociación con fármacos anticrisis sin lograr control adecuado. A los 5 años ingresó en una unidad de epilepsia de referencia para valorar abordaje quirúrgico. Sin embargo, se decidió abordaje conservador debido a que las valoraciones realizadas sugerían una dominancia izquierda del lenguaje. En años sucesivos presentó múltiples ingresos por descompensaciones, observándose epilepsia parcial continua, empeoramiento de la hemiparesia y atrofia progresiva en neuroimagen de control. Finalmente, en 2023, se realizó nueva valoración prequirúrgica en la que evaluaciones neuropsicológicas y la resonancia sugerían dominancia derecha del lenguaje, interviniéndose quirúrgicamente de hemisferotomía izquierda. **Resultados**: Tras la cirugía no ha vuelto a presentar crisis clínicas, aunque mantiene tratamiento en biterapia tras observarse actividad epileptiforme en registros electroencefalográficos sucesivos. **Conclusiones**: Dada la refractariedad de las crisis, las importantes secuelas que asocian y los buenos resultados observados, debemos plantearnos en estos pacientes el abordaje quirúrgico tras el uso inicial de fármacos anticrisis e inmunoterapia.

**Meningitis de Repetición: A Propósito de un Caso**

Saskia Vigni^1^, Violeta González Coello^1^, María Teresa Florido Capilla^1^, Sandra Rodríguez Martín^1^, Alba Jiménez Barreto^1^, María del Rosario Ríos Cejas^1^

^1^Servicio de Neurología, Hospital Universitario Nuestra Señora de Candelaria, 38010 Santa Cruz de Tenerife, España

**Introducción**: Hacer una revisión del manejo de la meningitis bacteriana recurrente en relación con un caso. **Material y Métodos**: Revisión de la historia clínica de un paciente con meningitis recurrente y de la literatura. **Resultados**: El paciente tuvo tres ingresos en UMI y neurología entre los años 2017 y 2024 por meningitis bacterianas por neumococo, que se resolvieron con el tratamiento antibiótico. En el segundo de ellos, en el año 2023, dado el ingreso previo se amplió el estudio etiológico, descartando inmunodeficiencias, y objetivando en el estudio dirigido una fístula de LCR en el techo de la fosa nasal izquierda. Se comentó el caso con neurocirugía y otorrinolaringología, estableciendo por su parte una actitud expectante inicialmente. Sin embargo, el paciente ingresa de nuevo en 2024 por un nuevo episodio de meningitis, similar a los previos. Se realizó una resonancia cerebral y de senos paranasales, confirmándose la persistencia de un meningocele en la fosa nasal izquierda en relación a la fístula de LCR descrita. Finalmente se realiza la reparación quirúrgica en julio de 2024, sin nuevos episodios posteriores. **Conclusión**: En los pacientes con meningitis recurrentes siempre se debe valorar la posibilidad de un defecto anatómico con un estudio dirigido, siendo preciso en ese caso el tratamiento quirúrgico habitualmente.

**Código Retina: A Propósito de 2 Casos**

Sofía del Águila Romero^1^, David Alejandro Padilla León^1^, Marcos Millet Oval^1^, Paloma Isabel Dupuy^1^, Vanessa Pallarés Santos^1^, Alba Elisa Bartolomé Yumar^1^, Ismael Naim Owrang Calvo^1^, José Antonio Rojo Aladro^1^

^1^Servicio de Neurología, Hospital Universitario de Canarias, 38320 San Cristóbal de La Laguna, España

**Introducción**: La oclusión de arteria central de la retina (OACR) es una urgencia oftalmológica que se manifiesta como pérdida visual súbita, indolora y monocular. Dada su similitud con un ictus isquémico, requiere un abordaje coordinado entre oftalmología y neurología para optimizar el pronóstico visual y realizar un correcto estudio etiológico. **Material y Método**: Casos clínicos. **Resultados**: Ambos pacientes consultaron por pérdida visual aguda en un ojo, sin otra focalidad neurológica. Fueron valorados inicialmente por oftalmología, que diagnosticó OACR no arterítica con menos de 4.5 horas de evolución. Fueron derivados a neurología y se activó protocolo de Código Ictus. Se realizó TC/Angio-TC craneal para descartar patología aguda asociada y se inició fibrinólisis intravenosa (FIV), seguido de ingreso en Unidad de Ictus para estudio etiológico. Ambos completaron tratamiento con oxigenoterapia hiperbárica. El primer caso fue un varón de mediana edad con factores de riesgo vascular y sin hallazgos en las pruebas complementarias relevantes, tratado con doble antiagregación durante 21 días y 8 sesiones de oxigenoterapia. El segundo, un paciente joven con antecedentes oncológicos y una fuente cardioembólica menor, con indicación de anticoagulación pero que tras valorar riesgo-beneficio por riesgo hemorrágico, se decidió doble antiagregación por 3 meses y 7 sesiones de oxigenoterapia. **Conclusiones**: La evidencia sobre el manejo agudo de la OACR es limitada, pero la FIV precoz parece ser más eficaz que el resto de las terapias. El abordaje multidisciplinar y el reconocimiento temprano de la clínica son clave para maximizar la recuperación visual, aunque el pronóstico funcional suele ser malo.

